# *CCN6* mutation detection in Chinese patients with progressive pseudo‐rheumatoid dysplasia and identification of four novel mutations

**DOI:** 10.1002/mgg3.1261

**Published:** 2020-04-29

**Authors:** Yingjie Wang, Ke Xiao, Yuemei Yang, Zhihong Wu, Jin Jin, Guixing Qiu, Xisheng Weng, Xiuli Zhao

**Affiliations:** ^1^ Department of Orthopedic Surgery Peking Union Medical College Hospital Peking Union Medical College & Chinese Academy of Medical Science Beijing China; ^2^ Department of Orthopedic Surgery West China Hospital Sichuan University Chengdu China; ^3^ Central Laboratory Peking Union Medical College Hospital Peking Union Medical College & Chinese Academy of Medical Science Beijing China; ^4^ Department of Medical Genetics School of Basic Medicine Peking Union Medical College Beijing China

**Keywords:** *CCN6*, novel mutations, progressive pseudo‐rheumatoid dysplasia, whole exome sequencing

## Abstract

**Background:**

No formal diagnostic criteria for progressive pseudo‐rheumatoid dysplasia (PPD) are available because of insufficient clinical data, which results in that PPD is often misdiagnosed with other diseases. Whole exome sequencing (WES) and Sanger sequencing were employed to reveal the novel mutations on *CCN6* of five patients with PPD from China in order to increase the clinical data of PPD.

**Methods:**

Four suspected PPD pedigrees containing five patients in total were collected from 1998 to 2018 in our medical center. The phenotypes of each suspected PPD case were recorded in detail, and peripheral blood samples were collected for subsequent sequencing. Genomic DNA was extracted from peripheral blood samples, and Agilent liquid phase chip capture system was utilized for efficient enrichment of whole exome region DNA. After acquiring raw sequenced reads of whole exome region, bioinformatics analysis was completed in conjunction with reference or genome sequence (GRCh37/hg19). Sanger sequencing was performed to identify the results of WES.

**Results:**

In total, four novel PPD‐related mutation sites in *CCN6* gene were identified including (*CCN6*):c.643 + 2T>C, (*CCN6*):c.1064_1065dupGT(p.Gln356ValfsTer33), (*CCN6*):c.1064G > A), and exon4:c.670dupA:p.W223fs.

**Conclusion:**

Our findings increase the clinical data of PPD including the *CCN6* mutation spectrum, the clinical symptoms and signs. Moreover, the study highlights the utility of WES in reaching definitive diagnoses for PPD.

## Graphical abstract

Whole exome sequencing is a time‐ and cost‐effective method to identify the gene mutations for some rare diseases including PPD. Four novel mutations on *CCN6* gene were first reported. Rare symptoms and signs of PPD patients were recorded and further increased physician's awareness of this disease.

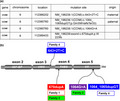

## 1 INTRODUCTION

Progressive pseudo‐rheumatoid dysplasia (PPD, OMIM 208,230), a rare autosomal recessive genetic disease (Warman et al., 2011; Wynne‐Davies, Hall, & Ansell, 1982), was first described by Wynne‐Davies et al. (1982). PPD is caused by the functional loss or abnormality of cellular communication network factor 6 (*CCN6*). The estimated prevalence rate of PPD is 1/1,000,000 in the United Kingdom but varies regionally worldwide. The prevalence rates reported in the Middle East and Gulf States, Turkey, India, and other endogamic communities are markedly higher than that of the United Kingdom (Bhavani et al., 2015; Dalal et al., 2012; Delague et al., 2005; Rai et al., 2016); however, only a few cases of PPD have been reported in East Asia.

Affected children are typically healthy at birth and develop normally during the first years of life. The onset of PPD usually occurs between the ages of 3 and 8 years with progressive stiffness, pain and deformities of multiple joints, and abnormal gait albeit without systemic and synovial inflammation. However, some patients may be asymptomatic until the age of 16 years or develop clinical manifestations as early as in the first years of life (Dalal et al., 2012; Delague et al., 2005; Gao, Ding, & Zhang, 2013; Montane et al., 2016; Neerinckx et al., 2015; Rai et al., 2016; Temiz et al., 2011). Generally, most of the joints are involved symmetrically after more than 10 years of disease development. First, the interphalangeal joints become affected, followed by the knees and hips, and disability rarely manifests at the cervical spine, elbows, wrists, and shoulders. However, the movement of almost all of the involved joints becomes limited over time (Garcia Segarra et al., 2012). Walking difficulties gradually develop until the patients lose autonomous mobility, usually in the second decade of life. The involved joints present as swollen, painful, stiff, of limited mobility, and with deformity. The most common initial symptoms of PPD include pain and bony enlargement of interphalangeal joints, ultimately leading to camptodactyly. Skeletal changes become increasingly apparent with time and are responsible for the flexion deformities, such as hips and knees. Scoliosis and short trunk can also be observed (Delague et al., 2005; Gao et al., 2013; Luo et al., 2015; Madhuri, Santhanam, Rajagopal, Sugumar, & Balaji, 2016; Sun et al., 2012; Yan et al., 2016; Ye et al., 2012; Zhou et al., 2007). Based on clinical findings, bone X‐ray examination should be performed first on suspicion of PPD, and the diagnosis can be confirmed through whole exome sequencing (WES). To date, more than 70 different mutations of the *CCN6* have been reported (Torreggiani et al., 2019).

In this study, the genetic characterization of four multiplex Chinese pedigrees displaying similar uncharacterized skeletal dysplasia was confirmed using WES and subsequent Sanger sequencing. Specifically, we identified four novel mutations in the *CCN6* (HGNC ID: 12,771) in five affected individuals. In addition, some rare clinical features, such as flexion deformity of elbows, were also reported. Overall, the aim of this study was to highlight some rare clinical features, radiographic features, and novel mutations of PPD to increase the awareness of this disorder among clinicians, thereby avoiding inappropriate treatment (such as antirheumatic treatment) and helping to relieve the associated pain and disability to improve the quality of life of the patient.

## 2 MATERIALS AND METHODS

### 2.1 Ethical compliance

The study protocol was approved by the Ethics Committee of Peking Union Medical College Hospital (PUMCH). All the experiments were performed in accordance with relevant guidelines and regulations.

### 2.2 Patient identification and pedigree establishment

Four suspected PPD pedigrees containing five patients in total were collected from 1998 to 2018 in PUMCH. The phenotypes of each suspected PPD case were recorded in detail from the time the patients were admitted to PUMCH. The first symptom of all five patients appeared between the ages of 3 and 8 years, whereas no symptoms were noted in infancy.

**Figure 1:**
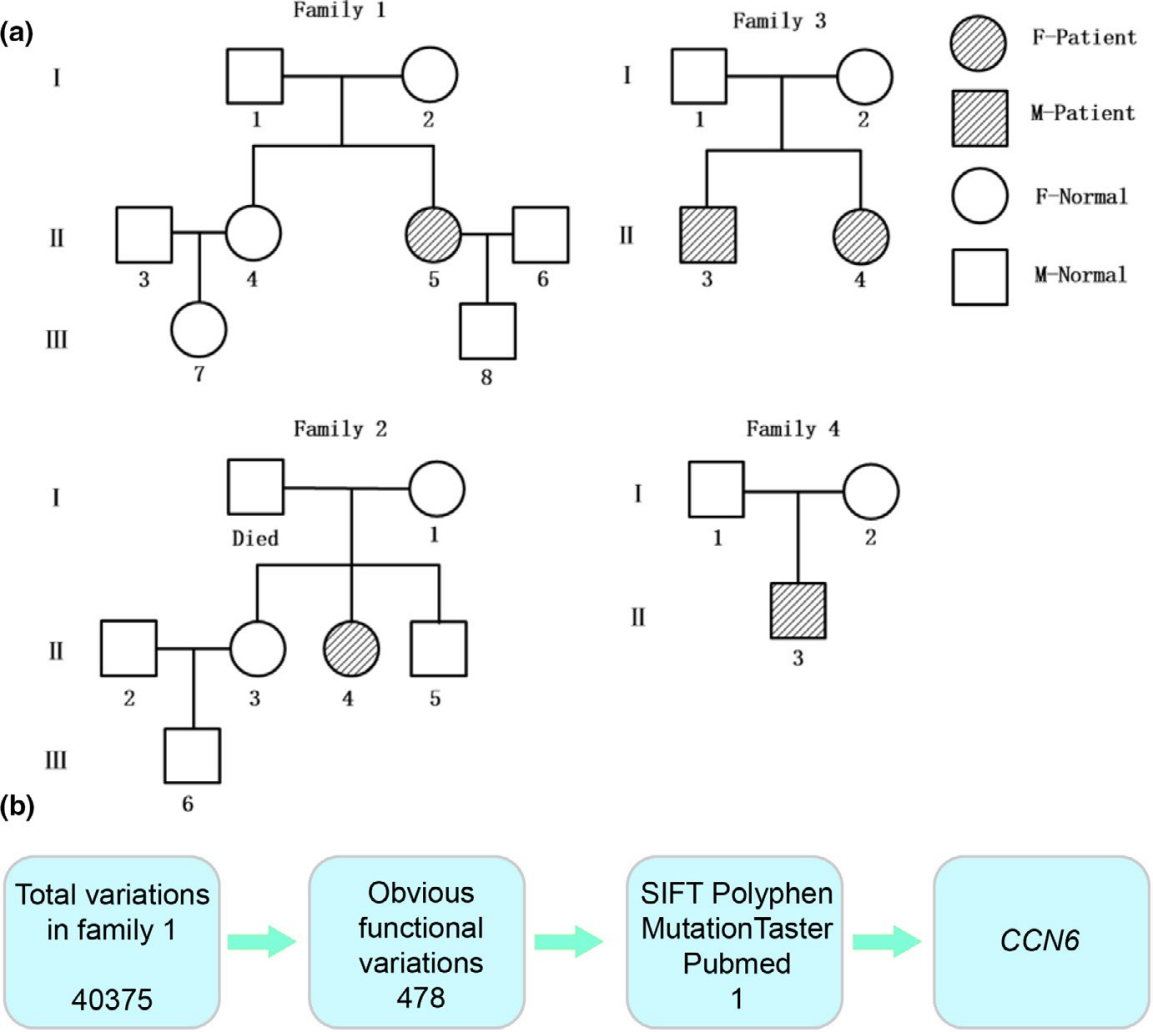
(a) Four pedigrees with suspected PPD. (b) The process identifying *CCN6* as the pathogenic gene of the patients with suspected PPD

Pedigree 1, originating from Hunan province of China, comprised one proband and seven other family members across three generations (four males, four females, age 5–54 years) (Figure 1a. Family 1). The other three pedigrees contained three to seven family members. Pedigree 3 included two probands who were siblings; a brother (age 23) and sister (age 17) (Figure 1a. Family 3).

### 2.3 Sample collection

Peripheral blood samples were collected from four pedigrees in ethylenediaminetetraacetic acid‐coated BD Vacutainer tubes (Becton Dickinson). Genomic DNA was extracted from peripheral blood samples using a QIA amp DNA Blood Mini Kit (Thermo Fisher) according to the manufacturer's protocol. Agarose gel electrophoresis was performed to analyze the degradation level of DNA and detect possible RNA or protein contamination. Qubit 4 (Thermo Fisher) was used for the precise quantification of extracted DNA.

### 2.4 Whole exome sequencing

Next‐generation sequencing, especially WES, is of considerable value for the diagnosis of genetic diseases as exon mutations underlie > 85% of all genetic diseases related to DNA mutations (Zhou et al., 2007). In the present study, the Agilent liquid phase chip capture system (Agilent Systems) was utilized for efficient enrichment of whole exome region DNA, for which DNA samples exceeding 0.6 µg total yield were chosen to create a database. Database building and capture assay was performed using the Agilent Sure Select Human All Exon V6 kit. WES was performed on the Illumina platform (U.S.) following quality inspection.

### 2.5 Bioinformatics analysis

After acquiring raw sequenced reads, bioinformatics analysis was completed in conjunction with reference or genome sequence (GRCh37/hg19, NC_000006.12). The process mainly consisted of three steps based on Sorting Intolerant From Tolerant (SIFT), PolyPhen2, and Mutation Taster software: step 1, quality evaluation of the sequencing data including analysis of the sequencing error rate along, sequencing depth and coverage, and the comparative rate; step 2, variation testing; step 3, variation screening and disease correlation prediction (Figure 1b).

### 2.6 Sanger sequencing

To verify the reliability of the WES results, 22 samples, independent from the samples for WES, were obtained from all members in the four pedigrees and applied to Sanger sequencing by the sequencing instrument (Applied Biosystems Inc.). Sequence analysis was performed using Chromas software (Version 2.6.6, Technelysium Pty. Ltd.). To identify mutation sites, sequencing results were compared with reference sequence, along with their parents’ sequences. The genotype was obtained by analyzing the type of bases at the mutation sites.

## 3 RESULTS

In total, five patients (three females and two males) were diagnosed clinically with PPD. The average age at onset of PPD was 5.4 years (3–8 years) and at diagnosis was 19 years (16–25 years). Among all enrolled patients with PPD, the diagnosis was supported by clinical features, radiological features, and laboratory findings.

**Figure 2:**
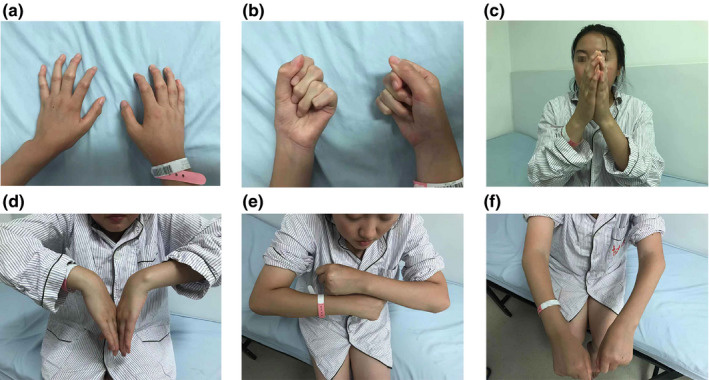
Photographs of the clinical features of a 25‐year‐old woman from pedigree 2. (a‐c) Swelling and deformity of the interphalangeal joints. (d) Reduced range of motion of the wrist joints. (e‐f) Flexion deformity of the elbow joints

**Figure 3:**
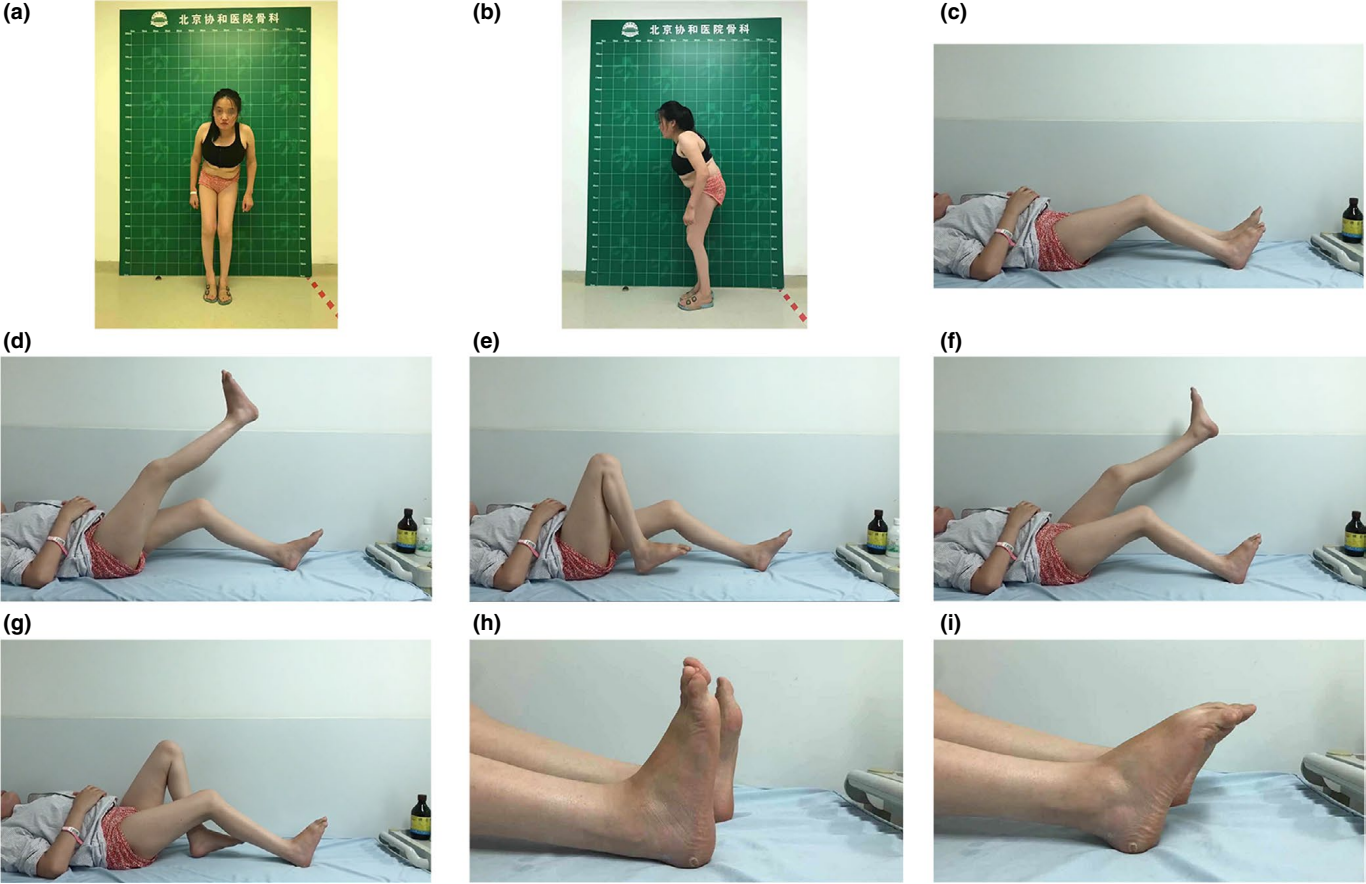
Photographs of the clinical features of a 25‐year‐old woman from pedigree 2. (a‐b) Overview of the hips, knees, and ankles. (c) Flexion deformity of the knee joints. (d‐g) Reduced range of motion of the bilateral knee joints. (h‐i) The limitation of ankle movement, a feature unique to PPD

**Figure 4:**
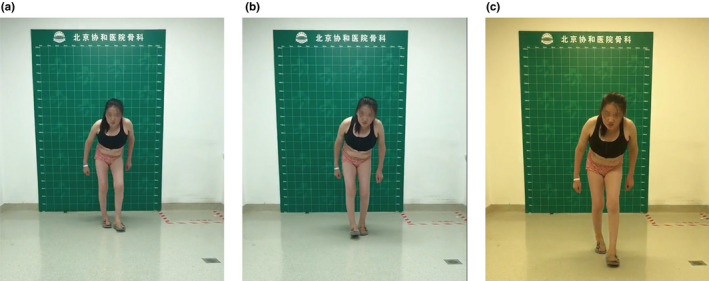
Photographs of the clinical features of a 25‐year‐old woman from pedigree 2. (a‐c) The claudication gait

The initial symptoms in most enrolled patients with PPD were stiffness and swelling of interphalangeal joints (Figure 2a‐c). Interestingly, the first symptom of one patient from pedigree 2 was the enlargement and deformity of knees at 2 years of age (Figure 3c‐g). The same patient presented with some rare clinical features including the flexion deformity of elbow joints (Figure 2e‐f) and the decreased range of motion of wrist (Figure 2d) and ankle joints (Figure 3h‐i). At the time of diagnosis, the most severe symptoms of the patients were difficulty walking, limping, and unequal length of both lower limbs (Figure 3a,b and Figure 4a‐c).

**Figure 5:**
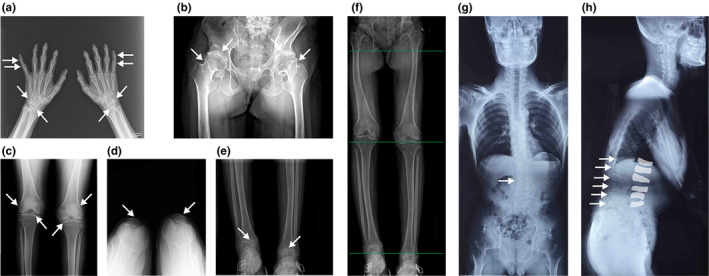
Photographs of the radiological features of a 25‐year‐old female from pedigree 2 (a‐f) and a 17‐year‐old male from pedigree 4 (g‐h). (a) Classical epiphyseal swelling of the interphalangeal joints with stiffness but without pain, along with narrow joint space of the wrists (white arrows). (b) Enlarged metaphysis of the femoral head and short and wide femoral necks (white arrows). (c) Enlarged tibial metaphysis (white arrows). Reduced joint space of the hips (b), knees (c), patellofemoral joints (d), and ankles (e). (f) Shorter right lower limb than left. Scoliosis (g) and initial flattening of some thoracolumbar vertebral bodies (h) (white arrows)

Garcia Segarra N et al. reported metaphyseal enlargement of the interphalangeal joints as an early radiological finding (Garcia Segarra et al., 2012). In the present study, radiological features consisted of irregular narrow interphalangeal (four patients, Figure 5a), wrist (one patients, Figure 5a), hip (four patients, Figure 5b), knee (three patients, Figure 5c‐d), and ankle (one patients, Figure 5e) joint spaces, along with scoliosis and platyspondylia (three patients) (Figure 5g,h). Enlarged metaphysis of the femoral head, short and wide femoral necks, and enlarged tibial metaphysis can be seen in Figure 5b,c. Different lengths of lower limbs owing to articular dysplasia are apparent in Figure 3a,b and Figure 5f. Most radiological changes in the five patients were in accordance with those in other case reports (el‐Shanti, Omari, & Qubain, 1997; Madhuri et al., 2016; Spranger, Albert, Schilling, Bartsocas, & Opitz, 1983; Teebi & Al Awadi, 1986; Temiz et al., 2011; Wynne‐Davies et al., 1982). Additionally, the inflammatory parameters of five patients are as follows: normal c‐reactive protein (CRP) and erythrocyte sedimentation rate (ESR) levels; negative rheumatoid factor (RF) and autoantibodies such as anti‐nuclear antibodies, anti‐cyclic citrullinated peptide (CCP) antibody, and anti‐citrullinated protein antibodies.

**Figure 6:**
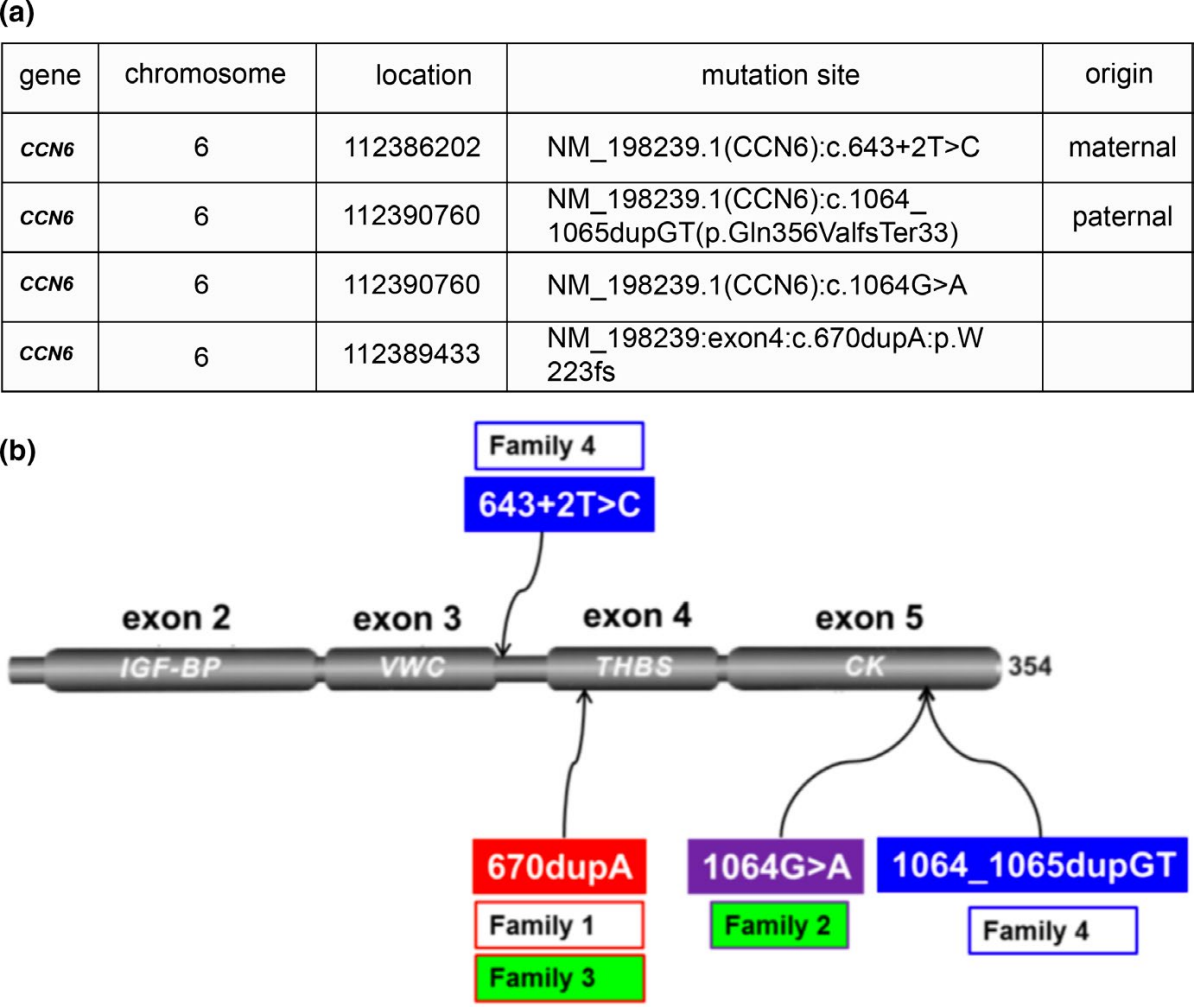
(a) Four novel mutation sites of *CCN6* and corresponding families. (b) Sanger verification for *CCN6* mutation sites and the distribution of four novel mutations on *CCN6*

**Figure 7:**
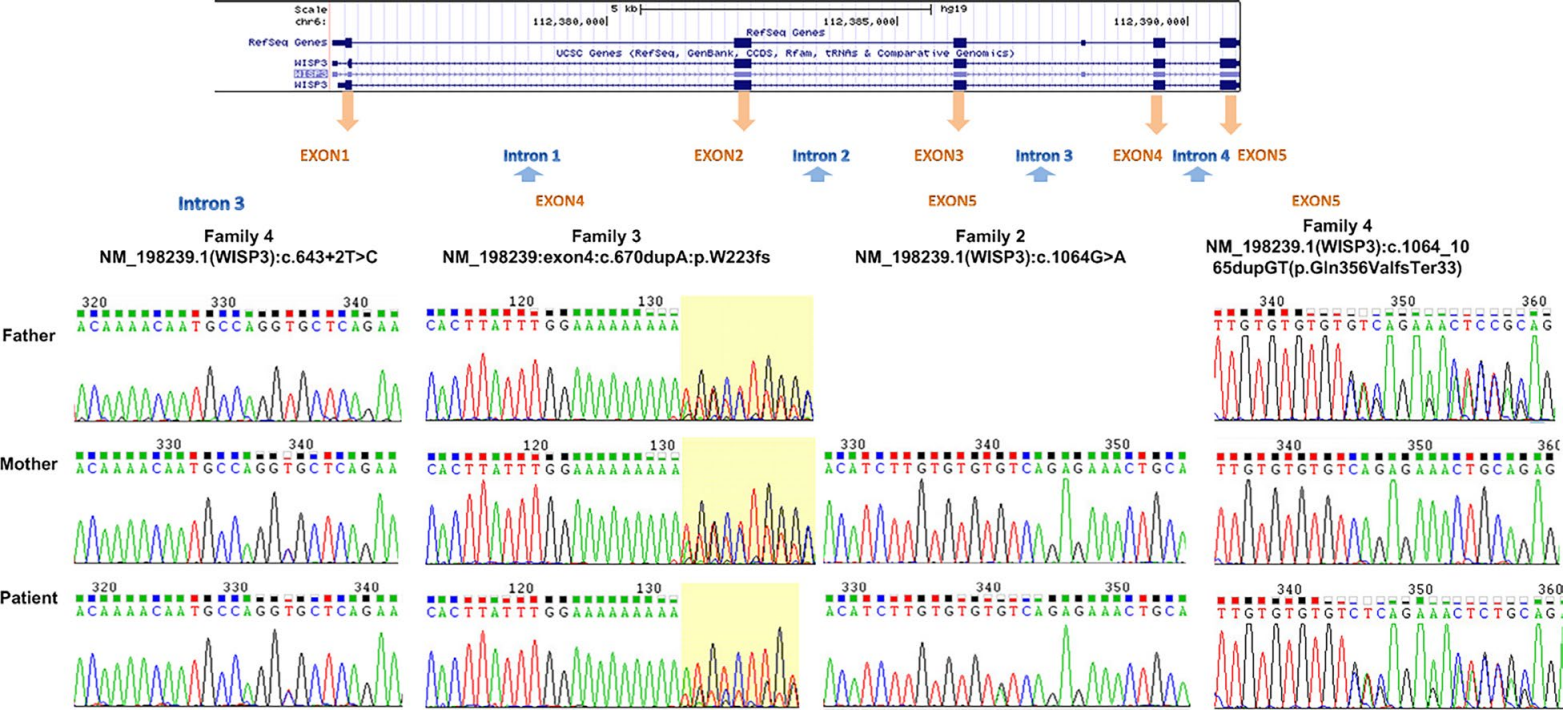
Sanger sequencing results for the various pedigrees

Based on the SIFT, PolyPhen2, and Mutation Taster software recommended by American College of Medical Genetics, 40,375 gene variations were discovered after sequence data screening. Subsequently, 478 functional variations were detected by deep processing and bioinformatics sequencing alignment. Through PubMed searches and Genemania software prediction, we identified *CCN6* as the pathogenic gene of the patients with PPD (Figure 1b). Finally, four previously unreported PPD‐related mutation sites in the *CCN6* were identified (Figure 6a and Figure 6b) which comprised NM_198239.1 (*CCN6*):c.643 + 2T>C, NM_198239.1 (*CCN6*):c.1064_1065dupGT(p.Gln356ValfsTer33), NM_198239.1(*CCN6*):c.1064G > A, and NM_198239:exon4:c.670dupA:p.W223fs. Notably, the patient in pedigree 4 harbored a novel compound heterozygous mutation in *CCN6* (NM_198239.1) including maternal NM_198239.1(*CCN6*):c.643 + 2T>C and paternal NM_198239.1(*CCN6*):c.1064_1065dupGTfsTer33 from heterozygous parents. In addition, NM_198239:exon4:c.670dupA:p.W223fs (frameshift mutation) and NM_003880:exon5:c.616dupA:p.W205fs (frameshift mutation) were identified as a novel compound heterozygous mutation of the *CCN6* in pedigrees 1 and 3. All the mutations were located in different regions (Figure 6b). The results of Sanger sequencing are shown in Figure 7. The results of Sanger sequencing were consistent with the result of WES, and further supported that the four mutation sites mentioned above were novel and crucial to the onset of PPD.

## 4 DISCUSSION

Currently, no formal diagnostic criteria for PPD are available. PPD constitutes a rare genetic disorder that is often misdiagnosed with other diseases such as juvenile idiopathic arthritis, myopathy, and Stickler's syndrome (Torreggiani et al., 2019). Fortunately, as the number of case reports continued to increase and through summarizing the clinical features, radiological features and laboratory findings of 81 patients with suspected PPD, Garcia Segarra N et al. defined “typical PPD” as the concomitant presence of onset in early childhood, stiffness and pain in multiple joints, enlarged interphalangeal joints, normal inflammatory parameters, and the absence of extraskeletal manifestations (Garcia Segarra et al., 2012). The radiological criteria for “typical PPD” include metaphyseal enlargement of the interphalangeal joints, reduced articular space with large dysplastic epyphyses at the hip and knee joints, platyspondyly with the anterior breakage of vertebral bodies, the absence of articular bone erosions, and generalized osteopenia starting in adolescence (Garcia Segarra et al., 2012).

Four pedigrees were enrolled in the present study based on the typical clinical and radiological features of five probands who presented with multiarticular involvement, such as swollen metaphysis, deformity, reduced range of motion, and narrow joint space of the interphalangeal joints, wrists, elbows, hips, knees, and ankles. Aside from abnormal gait, the lack of systemic inflammation, as evidenced by normal RF, CCP, ESR, and CRP, also supported the diagnosis of PPD. All the symptoms and signs found in the five probands were consistent with those from published studies (Cassa et al., 2016; Delague et al., 2005; Garcia Segarra et al., 2012; Luo et al., 2015; Rai et al., 2016), in which *CCN6* was the pathogenic gene for PPD. There may be other pathogenic genes attributing to PPD. Compared with targeted sequencing, WES could find new pathogenic genes (apart from *CCN6*) and/or new mutation sites on the *CCN6*. In this study, four novel mutation sites on *CCN6* were found through WES.

*CCN6,* located on chromosome 6q22 (Hurvitz et al., 1999), encodes a signaling factor essential for cartilage homeostasis that is a member of growth factors of the CTGF, cyr61/cef10, nov (*CNN*) gene family that regulate cell proliferation, cell differentiation, and cell migration in connective tissue (Hurvitz et al., 1999; Katsube, Sakamoto, Tamamura, & Yamaguchi, 2009; Zuo et al., 2010). Only biallelic loss‐of‐function mutations in the *CCN6* are reported to result in PPD. Only mutations on one gene of a pair of allele, including parents and siblings, are not reported to present any articular symptom or signs (Garcia Segarra et al., 2012; Hurvitz et al., 1999; Madhuri et al., 2016).

Notably, the frequency of the different mutations in the *CCN6* vary geographically. For example, c.156C>A mutations are frequently found in areas such as Lebanon, Italy, Syria, France, and Turkey, with the most frequent mutations in India being c.1010G>A and c.233G>A (Bhavani et al., 2015; Dalal et al., 2012; Delague et al., 2005; Garcia Segarra et al., 2012; Hurvitz et al., 1999; Madhuri et al., 2016; Temiz et al., 2011). However, no correlations have been observed between genotypes and phenotypes. Even siblings with the same mutations may present different clinical features (Bhavani et al., 2015; Dalal et al., 2012; Delague et al., 2005; Ekbote et al., 2013; Hurvitz et al., 1999; Sun et al., 2012). Nevertheless, mutations in the *CCN6* do not appear to affect intelligence and facial appearance and no extraskeletal manifestations have been reported (Rai et al., 2016; Sailani et al., 2018; Taspinar, Kelesoglu, Keskin, & Uludag, 2016; Yan et al., 2016).

In the present study, four novel mutations in the *CCN6* were revealed by WES and confirmed by Sanger sequencing in four multiplex pedigrees displaying similar uncharacterized skeletal dysplasia. These mutations comprised NM_198239.1(*CCN6*):c.643 + 2T>C, NM_198239.1(*CCN6*):c.1064_1065dupGTfsTer33, NM_198239.1(*CCN6*):c.1064G > A, and NM_198239:exon4:c.670dupA:p.W223fs. The four novel mutation sites were predicted to be crucial to the onset of PPD. Specifically, the T to C transduction in intronic DNA occurred two bases away from *CCN6* cDNA base 643 in the exon–intron boundary, which constitutes sequence necessary for recognition by the splicing machinery. Such splicing mutations are prone to posttranscriptional RNA splicing error, thus leading to protein changes and, for *CCN6*, the likely onset of PPD. The GT duplication causes the subsequent substitution of glutamine to valine at position 356 in the *CCN6* protein sequence with the frameshift altering the subsequent amino acids as well, thereby likely disrupting protein function. Similar protein disruption was considered to result from the G to A transition in exon 5 and the A duplications.

Through combination of the sequencing results with the complete clinical documentations, five patients in the four pedigrees were diagnosed with PPD, supporting that WES combined with Sanger sequencing can be of considerable assistance in disease screening for families with hereditary disease. The novel mutation sites on the *CCN6* elucidated in our study may further enhance our understanding of the correlation between *CCN6* protein function and PPD pathogenesis. Our study revealed the genotype of patient with PPD using WES technology in Chinese pedigrees, and our results underscored the utility of WES in arriving at a definitive diagnosis for rare skeletal dysplasia.

It has been shown in vitro that *CCN6* increases the expression level of collagen type II, aggrecan, and the transcription factor SOX9, stimulates superoxide dismutase activity (Davis, Chen, & Sen, 2006; Miller & Sen, 2007; Sen, Cheng, Goldring, Lotz, & Carson, 2004), and inhibits cell proliferation and viability in chondrocytes (Wang et al., 2013). In addition, *CCN6* promotes the precursor cell differentiation of human chondrocytes (Wang et al., 2013). Moreover, chondrocyte culture of a patient with PPD showed more rapid proliferation and an abnormal amount of matrix metalloproteinase (Zhou et al., 2007), and the matrix metalloproteinase level was similar to that observed for osteoarthritis chondrocyte culture (Baker et al., 2012). It was also reported that the knock‐out or down‐expression of *CCN6* did not lead to any pathologic changes in mice, whereas *CCN6* over‐expression downregulated bone morphogenetic protein and Wnt pathways in zebra fish (Hann, Kvenvold, Newby, Hong, & Warman, 2013; Nakamura, Cui, Fernando, Kutz, & Warman, 2009). However, the lack of *CCN6* expression in patients with PPD did not appear to markedly influence such pathways. Alternatively, hyperproliferative chondrocytes consequent to low *CCN6* expression levels may explain the enlarged metaphysis in patients. In addition, the progressive narrowing of all joint spaces and reduced joint mobility may be due to delayed differentiation of chondrocyte precursor cells, with concomitant reduced collagen type II synthesis and secretion to the extracellular matrix (Bhavani, Shah, Shukla, Dalal, & Girisha, 1993; Zhou et al., 2007).

The clinical features of PPD overlap those of juvenile rheumatoid arthritis, mucopolysaccharidosis, and ankylosing spondylitis. Although the symptoms of PPD may appear in early childhood, patients with PPD are often misdiagnosed or receive a delayed diagnosis until joints and spine deformities become noticeable (Ekbote et al., 2013; Garcia Segarra et al., 2012; Taspinar et al., 2016; Torreggiani et al., 2019). Thus, affected individuals may not receive optimal medical management. Early detection and diagnosis of PPD are therefore important for avoiding unnecessary anti‐inflammatory and immunosuppressive treatments and facilitating relief from the pain and disability associated with PPD. However, the current treatment for PPD is merely supportive and based on pain medication, physical therapy, and surgical interventions (Isidor et al., 2012; Jurgens et al., 2015; Torreggiani et al., 2019).

In conclusion, the findings of our study increased the evidence to support timely diagnosis and treatment of PPD. In particular, the results demonstrated that WES was helpful to ascertain *CCN6* mutations before further screening the genotypes of patients and other members of their family. Overall, the five probands presented similar clinical phenotypes but no specific correlation between genotypes and phenotypes was detected. Nevertheless, the results of the study expanded the *CCN6* mutation spectrum that is associated with PPD and may aid in further elucidating the function of *CCN6*. In the future, it will be necessary to establish and perfect the diagnostic criteria for PPD. Additionally, more effective and cost‐effective gene panel sequencing should be validated in order to make a definite diagnosis of PPD based on typical signs and symptoms combined with the results of gene panel sequencing. Finally, additional attention should be paid to developing effective and efficient treatments for PPD, such as gene therapy and targeted biologics.

## CONFLICT OF INTEREST

The authors declare that there is no conflict of interest.

## AUTHOR CONTRIBUTIONS

Yingjie Wang and Ke Xiao were involved in preparation of the research images, review of the literature, and writing the paper. Yuemei Yang and Jin Jin were involved in collection of the data and some data analysis. Zhihong Wu, Qiu Guixing, Xiuli Zhao, and Xisheng Weng were involved in provision of research ideas and review of the paper. All authors have seen and agree with the contents of the manuscript.

## INFORMED CONSENTS

Written informed consents for publication of this research and accompanying images were obtained from all participants or, if the subjects were under 18 years of age, from a parent or legal guardian.

## Data Availability

The data may be available from the corresponding author upon reasonable request.
